# Analysis of the genome sequence of *Phomopsis longicolla*: a fungal pathogen causing Phomopsis seed decay in soybean

**DOI:** 10.1186/s12864-017-4075-x

**Published:** 2017-09-05

**Authors:** Shuxian Li, Omar Darwish, Nadim W. Alkharouf, Bryan Musungu, Benjamin F. Matthews

**Affiliations:** 1 United States Department of Agriculture, Agricultural Research Service (USDA-ARS), Crop Genetics Research Unit, Stoneville, MS 38776 USA; 2 Department of Computer and Information Sciences, Towson University, Towson, MD 21252 USA; 3 Department of Plant Biology, Southern Illinois University, Carbondale, IL 62901 USA; 4 Current address: USDA-ARS, Warm Water Aquaculture Unit, Stoneville, MS 38776 USA; 5 USDA-ARS, Soybean Genomics and Improvement Lab, Beltsville Agriculture Research Center, Beltsville, MD 20705 USA

**Keywords:** Genome, *Phomopsis longicolla*, Phomopsis seed decay, Soybean

## Abstract

**Background:**

*Phomopsis longicolla* T. W. Hobbs (syn. *Diaporthe longicolla*) is a seed-borne fungus causing Phomopsis seed decay in soybean. This disease is one of the most devastating diseases reducing soybean seed quality worldwide. To facilitate investigation of the genomic basis of pathogenicity and to understand the mechanism of the disease development, the genome of an isolate, MSPL10–6, from Mississippi, USA was sequenced, de novo assembled, and analyzed.

**Results:**

The genome of MSPL 10–6 was estimated to be approximately 62 Mb in size with an overall G + C content of 48.6%. Of 16,597 predicted genes, 9866 genes (59.45%) had significant matches to genes in the NCBI nr database, while 18.01% of them did not link to any gene ontology classification, and 9.64% of genes did not significantly match any known genes. Analysis of the 1221 putative genes that encoded carbohydrate-activated enzymes (CAZys) indicated that 715 genes belong to three classes of CAZy that have a direct role in degrading plant cell walls. A novel fungal ulvan lyase (PL24; EC 4.2.2.-) was identified. Approximately 12.7% of the *P. longicolla* genome consists of repetitive elements. A total of 510 potentially horizontally transferred genes were identified. They appeared to originate from 22 other fungi, 26 eubacteria and 5 archaebacteria.

**Conclusions:**

The genome of the *P. longicolla* isolate MSPL10–6 represented the first reported genome sequence in the fungal *Diaporthe-Phomopsis* complex causing soybean diseases. The genome contained a number of Pfams not described previously. Information obtained from this study enhances our knowledge about this seed-borne pathogen and will facilitate further research on the genomic basis and pathogenicity mechanism of *P. longicolla* and aids in development of improved strategies for efficient management of Phomopsis seed decay in soybean.

**Electronic supplementary material:**

The online version of this article (doi:10.1186/s12864-017-4075-x) contains supplementary material, which is available to authorized users.

## Background

*Phomopsis longicolla* T. W. Hobbs (syn. *Diaporthe longicolla*) is a seed-borne fungus primarily causing Phomopsis seed decay (PSD) in soybean, *Glycine max* (L.) Merrill [1–4]. This disease decreases seed quality and has been found in most soybean production areas, worldwide [2, 4, 5]. The common symptoms of PSD include discolored seed that are both shriveled and elongated or cracked seed coats and are chalky-white in appearance. Soybean seed infected by *P. longicolla* often lack of visible symptoms or signs at harvest [6]. It has been reported that soybean seed infected by *P. longicolla*, whether symptomless or having symptoms, could have very low seed germination, reduced seedling vigor, and poor stands [4, 7]. Poor seed quality of soybean could be due to the alteration of seed composition, reduction of oil quality, or moldy and/or split seed caused by *P. longicolla* [8]. PSD is one of the most economically important diseases of soybean. This disease has caused significant soybean yield loss. [9, 10]. If the environment is warm and humid during the late growing season from pod fill through harvest, it favors pathogen growth and PSD development [11].

Management of PSD is very challenging. Inconsistent reductions of PSD have been reported when common agronomic practices were used. Practices included crop rotation with non-host or non-legume crops, conventional tillage to reduce pathogen inoculum, and prompt harvest when soybeans matured to avoid late season wet weather [2]. In addition, fungicide treatments could be used as an option to reduce PSD and other soybean diseases. However, they were not always effective in controlling PSD [12–14]. Planting cultivars with resistance to PSD is a long-term strategy to manage PSD. In past decades, most research conducted on the host resistance, such as identifying resistance sources by screening soybean germplasms, commercial cultivars, and breeding lines [5, 15–17], breeding for resistant lines and cultivars [18], and investigating inheritance of resistance to PSD [19–21]. In addition, genetic mapping of resistance to PSD was reported [22]. However, information about the genomic features and mechanisms underlying the pathogenicity of *P. longicolla* on soybean were lacking. It is well-known that plant cell walls are the primary barrier against pathogen invasions. In order to infect plants, a plant pathogen should have the ability to pass through the plant cell wall. Plant cell wall degrading enzymes (PCWDEs) are a subset of carbohydrate-activated enzymes (CAZy) that are produced by plant pathogens to degrade plant cell walls. There was no information about PCWDEs in *P. longicolla.* Further, horizontal gene transfer or lateral gene transfer has been inferred to be the movement of genetic material between different organisms [23, 24]. If true it is a major force driving the evolution of both bacteria and eukaryotes [25, 26]. To date there was no report inferring the possibility of HGT in *P. longicolla.* Understanding the nature of the pathogen and mechanisms of PSD development in host plants will help us develop better disease management strategies. In recent years, genomic studies have made important contributions to research and disease management in plant pathology [27]. The next-generation sequencing technology has facilitated the genomics-based approached to both improve disease resistance in crops and enhance our understanding the mechanism of pathogenicity. The genomic approaches could provide an alternative way to identify host resistances.

To facilitate investigation of the genomic basis of pathogenicity in *P. longicolla* and to understand the mechanism of the disease development, the genome of isolate MSPL10–6 was sequenced and de novo assembled [28]. This research was conducted to analyze the genome sequences of the *P. longicolla* isolate MSPL10–6. The aims here were to understand the genome features of *P. longicolla*, identify genes encoding plant cell wall degrading enzymes, discover and classify the repeat elements in the genome, and investigate the potentially horizontally transferred genes in the *P. longicolla* genome.

## Results

### General genome features

The genome of the *P. longicolla* isolate MSPL 10–6 was assembled from both the paired-end and mate-pair libraries with the short oligonucleotide assembler package (SOAP), a denovo assembler. The oligonucleotides formed 108 scaffolds of 500 bases or larger. As reported in previous studies, the N50 length was 1,039,102 bp, and the largest scaffold contained 6,247,470 bp. The genome size was estimated to be approximately 62 Mb with an overall G + C content of 48.6% [28]. Statistics of genome sequencing and assembly are summarized in Table 1.

**Table 1:** Statistics of genome sequencing and assembly of *Phomopsis longicolla* isolate MSPL 10–6

Sequencing Statistics Library	Paired End (0.5 Kb inserts)	Mate Pair (3.9 Kb inserts)	Total
Raw data			
Size	6.9 Gb	16.2 Gb	23.1 Gb
Coverage	108 X	253 X	361 X
Processed data			
Size	6.2 Gb	8.2 Gb	14.4 Gb
Coverage	97 X	128 X	225 X
Assembly Statistics		Contigs	Scaffolds
Total assembly size		62 Mb	66.7 Mb
Total assembled sequences		12,329	108
Longest sequence length		215 Kb	6.2 Mb
Average sequence length		5054 bp	618 Kb
N90 index		2900	62
N90 length		3.21 Kb	299 Kb
N50 index		662	17
N50 length		26.3 Kb	1.04 Mb

### Gene prediction and annotation

Gene prediction analysis yielded a total of 16,597 genes (Average Length was 1704 bp, Total Length was 28,287,360 bp, Total Coding Length was 24,840,981 bp), of which 4334 genes where found to consist of a single exon (Average Length = 1219 bp). The total number of exons in all predicted genes was 47,213 (Average Length was 3622 bp, Total Length = 4,435,952 bp).

**Fig. 1:**
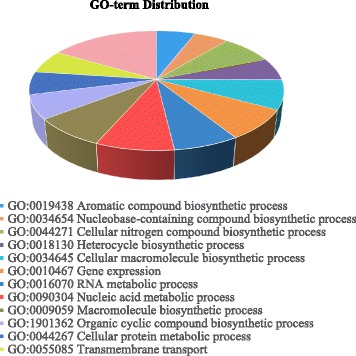
Functional categorization and distribution of potential genes in the genome of *Phomopsis longicolla* isolate MSPL 10–6

Of 16,597 genes predicted, 9866 genes (59.45%) had significant matches to genes in the NCBI nr database, while 18.01% of them did not link to any gene ontology (GO) classification. Further, 9.64% of the genes did not significantly match any known genes. Enzyme codes were assigned to 15.45% of the genes. The gene prediction statistics are summarized in Table 2. Functional categorization and distribution of potential genes in the *P. longicolla* genome are shown in Fig. 1.

**Table 2:** Statistics of genome annotation of *Phomopsis longicolla* isolate MSPL 10–6

Genes			
Single Exon Genes		All Genes	
Count	4334	Count	16,597
Average Length	1219.1	Average Length	1704.37
Median Length	1026	Median Length	1411
Total Length	5,283,582	Total Length	28,287,360
Average Coding Length	1216.07	Average Coding Length	1496.72
Median Coding Length	1023	Median Coding Length	1236
Total Coding Length	5,270,457	Total Coding Length	24,840,981
Average Score	0.82	Average Score	2.25
Total Score	3560.51	Total Score	37,365.46
Ave Exons Per	1	Ave Exons Per	2.84
Med Exons Per	1	Med Exons Per	2
Total Exons	4334	Total Exons	47,213
Exons			
Initial		Terminal	
Count	12,262	Count	12,262
Average Length	361.76	Average Length	636.18
Median Length	214	Median Length	434
Total Length	4,435,952	Total Length	7,800,818
Average Score	0.8	Average Score	0.79
Total Score	9765.44	Total Score	9696.51
Internal		Single	
Count	18,355	Count	4334
Average Length	399.55	Average Length	1216.07
Median Length	233	Median Length	1023
Total Length	7,333,754	Total Length	5,270,457
Average Score	0.78	Average Score	0.82
Total Score	14,343	Total Score	3560.51

### Plant cell-wall degrading enzymes

**Fig. 2:**
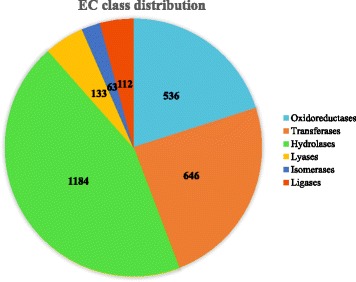
Enzyme code (EC) class distributions in the genome of *Phomopsis longicolla* isolate MSPL 10–6 based on the gene ontology classification

**Fig. 3:**
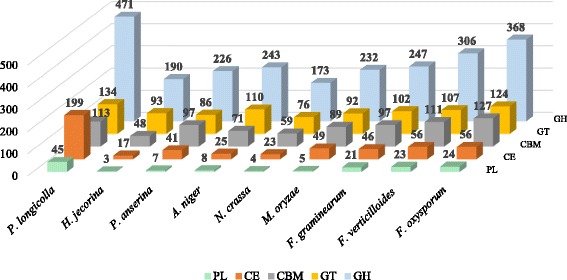
The number of different classes of the carbohydrate-activated enzymes (CAZy) in *Phomopsis longicolla* and other ascomycete fungi (*Aspergillus niger*, *Hypocrea jecorina, Fusarium oxysporum*, *F. virguliforme*, *F. verticilloides*, *Magnaporthe oryzae*, *Neurospora crassa*, and *Podospora anserine*)

The enzyme classification code (EC number) class distributions (level 3) are summarized in Fig. 2. Of 2674 EC enzyme-like orthologs identified, 1184 (44.3%) were related to hydrolases or hydrolytic enzyme, while 63 (2.4%) sequences were related to isomerases. An abundance of genes encoding plant cell-wall degrading enzymes (PCWDEs) were found in the *P. longicolla* genome. Of 1221 putative genes that encode carbohydrate-activated enzymes (CAZys), (Additional file 1: Table S1), 199 genes encoded carbohydrate esterases (CE), 471 encoded glycoside hydrolases (GH), and 45 encoded polysaccharide lyases (PL) (Table 2). In the CAZy family, enzymes that have the same substrate and description could have different “domain” structures and coding sequences, such as CE1 – CE5, GH 10 and GH11 (Table 3). Three other classes of CAZys with indirect roles on degrading carbohydrates were auxiliary activities (AAs), carbohydrate-binding modules (CBMs), and glycosyl-transferases (GTs). The number of putative genes identified in the AA, CBM, and GT classes were 259, 113, and 134, respectively (Table 4). Comparisons of the numbers of CAZys in *P. longicolla* with other Ascomycete fungi are shown in Fig. 3.

**Table 3:** A list of the carbohydrate-activated enzymes (CAZy) identified in the genome of *Phomopsis longicolla* isolate MSPL 10–6

CAZy family^a^	Substrate	Description	EC	Copy number
CE1	Hemicellulose (xylan)	Acetyl xylan esterase	3.1.1.72	33
		Feruloyl esterase	3.1.1.73	
CE2	Hemicellulose (xylan)	Acetyl xylan esterase	3.1.1.72	2
CE3	Hemicellulose (xylan)	Acetyl xylan esterase	3.1.1.72	10
CE4	Hemicellulose (xylan)	Acetyl xylan esterase	3.1.1.72	8
CE5	Hemicellulose (xylan)	Acetyl xylan esterase	3.1.1.72	18
	Cutin	Cutinase	3.1.1.74	
CE7	Hemicellulose (xylan)	Acetyl xylan esterase	3.1.1.72	1
CE8	Pectin (homogalacturonan)	Pectin methylesterase	3.1.1.11	6
CE9	Polysaccharides	N-acetylglucosamine 6-phosphate	3.5.1.25	1
		Deacetylase	3.5.1.80	
CE10	Polysaccharides	Arylesterase	3.1.1.-	105
CE12	Pectin(homogalacturonan, rhamnogalacturonan I)	Pectin acetylesterase	3.1.1.-	5
	Hemicellulose	Acetyl pectin esterase	3.1.1.72	
CE14	Polysaccharides	N-acetylglucosaminylphosphatidy-linositol deacetylase	3.5.1.89	1
CE15	Polysaccharides	4-O-methyl-glucuronoyl methylesterase	3.1.1.-	2
CE16	Polysaccharides	Acetylesterase	3.1.1.6	7
GH1	Cellulose	β-glucosidase	3.2.1.21	6
	Hemicellulose (xylan, xyloglucan)	β-xylosidase	3.2.1.37	
	Pectin (rhamnogalacturonan I)	β-galactosidase	3.2.1.23	
GH2	Hemicellulose (xylan, xyloglucan, galactomannan)	β-mannosidase	3.2.1.25	10
	Pectin (rhamnogalacturonan I)	β-glucuronidase	3.2.1.31	
GH3	Cellulose	β-glucosidase	3.2.1.21	23
	Hemicellulose	β-xylosidase	3.2.1.37	
	(xylan, xyloglucan)		3.2.1.74	
	Pectin	exo-β-1,4-glucanase		
GH5	Cellulose	endo-β-1,4-glucanase	3.2.1.4	27
	Hemicellulose (galactomannan)	endo-β-1,4-xylanase	3.2.1.8	
	Pectin (rhamnogalacturonan I)	exo-β-1,4-glucanase	3.2.1.74	
GH6	Cellulose	Cellobiohydrolase	3.2.1.91	4
		endo-β-1,4-glucanase	3.2.1.4	
GH7	Cellulose	endo-β-1,4-glucanase	3.2.1.4	9
		Cellobiohydrolase	3.2.1.176	
GH9	Cellulose	Cellusae	-	1
GH10	Hemicellulose (xylan)	endo-β-1,4-xylanase	3.2.1.8	7
GH11	Hemicellulose (xylan)	endo-β-1,4-xylanase	3.2.1.8	4
GH12	Cellulose	endo-β-1,4-glucanase	3.2.1.4	6
	Hemicellulose (xyloglucan)	Xyloglucanase	3.2.1.151	
GH13	Polysaccharides	α-amylase	3.2.1.1	17
GH15	Polysaccharides	Glucoamylase	3.2.1.3	1
GH16	Hemicellulose	Xyloglucanase	3.2.1.151	21
GH17	Polysaccharides	endo-1,3-β-glucosidase	3.2.1.39	7
GH18	Polysaccharides	Chitinase	3.2.1.14	27
		endo-β-N-acetylglucosaminidase	3.2.1.96	
GH20	Polysaccharides	β-hexosaminidase	3.2.1.52	3
GH26	Polysaccharides	beta-mannanase	3.2.1.78	1
GH27	Hemicellulose (xylan, xyloglucan, galactomannan)	α-galactosidase	3.2.1.22	2
		α-N-acetylgalactosaminidase	3.2.1.49	
GH28	Pectin (homogalacturonan, rhamnogalacturonan I)	Polygalacturonase	3.2.1.15	21
GH29	Oligosaccharides	alpha-L-fucosidase	3.2.1.51	4
GH30	Polysaccharides	Glucosylceramidase	3.2.1.45	4
GH31	Hemicellulose (xyloglucan)	α-xylosidase	3.2.1.177	7
GH32	Sucrose	Invertase	3.2.1.26	6
GH33	Oligosaccharides	exo-α-sialidase	3.2.1.18	1
GH35	Hemicellulose (xylan, xyloglucan, galactomannan)	β-galactosidase	3.2.1.23	7
	Pectin (rhamnogalacturonan I)	exo-β-1,4-galactanase	3.2.1.-	
GH36	Hemicellulose (xylan, xyloglucan, galactomannan)	α-galactosidase	3.2.1.22	1
		α-N-acetylgalactosaminidase	3.2.1.49	
GH37	Trehalose	α,α-trehalase	3.2.1.28	2
GH38	Oligosaccharides	α-mannosidase	3.2.1.24	1
GH39	Oligosaccharides	alpha-L-iduronidase	3.2.1.76	2
GH42	Oligosaccharides	beta-galactosidase	3.2.1.23	1
GH43	Hemicellulose (xylan)	β-xylosidase	3.2.1.37	40
	Pectin (rhamnogalacturonan I)	α-L-arabinofuranosidase	3.2.1.55	
GH45	Cellulose	endo-β-1,4-glucanase	3.2.1.4	2
GH47	Oligosaccharides	α-mannosidase	3.2.1.113	12
GH51	Cellulose	endo-β-1,4-glucanase	3.2.1.4	4
	Hemicellulose (xylan,xyloglucan)	β-xylosidase	3.2.1.37	
GH53	Pectin (rhamnogalacturonan I)	endo-β-1,4-galactanase	3.2.1.89	4
GH54	Pectin	alpha-L-arabinofuranosidase	3.2.1.55	1
GH55	Polysaccharides	endo-1,3-β-glucosidase	3.2.1.39	6
GH62	Polysaccharides	alpha-L-arabinofuranosidase	3.2.1.55	1
GH63	Oligosaccharides	α-glucosidase	3.2.1.106	4
GH64	Polysaccharides	endo-1,3-β-glucosidase	3.2.1.39	4
GH65	Polysaccharides	alpha,alpha-trehalase	3.2.1.28	2
GH67	Polysaccharides	alpha-glucuronidase	3.2.1.139	1
GH71	Polysaccharides	α-1,3-glucanase	3.2.1.59	9
GH72	Polysaccharides	β-1,3-glucanosyltransglycosylase	2.4.1.-	10
GH74	Cellulose	endo-β-1,4-glucanase	3.2.1.4	11
	Hemicellulose (xyloglucan)	Xyloglucanase	3.2.1.151	
GH76	Oligosaccharides	α-1,6-mannanase	3.2.1.101	14
GH78	Pectin	α-L-rhamnosidase	3.2.1.40	14
GH79	Pectin (rhamnogalacturonan I)	β-glucuronidase	3.2.1.31	9
GH81	Polysaccharides	endo-1,3-β-glucosidase	3.2.1.39	2
GH88	Polysaccharides	β-glucuronyl hydrolase	3.2.1.-	1
GH92	Oligosaccharides	Mannosyl-oligosaccharide alpha-1,2-mannosidase	3.2.1.113	8
GH93	Pectin (rhamnogalacturonan I)	exo-α-L-1,5-arabinanase	3.2.1.-	7
GH94	Cellulose	cellobiose phosphorylase	2.4.1.20	1
GH95	Hemicellulose (xyloglucan)	α-1,2-L-fucosidase	3.2.1.63	3
GH105	Pectin	Rhamnogalacturonyl hydrolase	3.2.1.172	8
GH106	Polysaccharides	alpha-L-rhamnosidase	3.2.1.40	4
GH109	Polysaccharides	α-N-acetylgalactosaminidase	3.2.1.49	20
GH114	Polysaccharides	endo-α-1,4-polygalactosaminidase	3.2.1.109	3
GH115	Hemicellulose (xylan)	Xylan α-1,2-glucuronidase	3.2.1.131	4
GH125	Oligosaccharides	exo-α-1,6-mannosidase	3.2.1.-	4
GH127	Oligosaccharides	β-L-arabinofuranosidase	3.2.1.185	3
GH128	Polysaccharides	endo-1,3-β-glucosidase	3.2.1.39	6
GH131	Cellulose	exo-β-1,3/1,4/1,6-glucanase	3.2.1.-	8
	Hemicellulose			
GH132	Polysaccharides	Activity on β-1,3glucan	–	2
GH133	Polysaccharides	amylo-α-1,6-glucosidase	3.2.1.33	1
GH134	Polysaccharides	endo-β-1,4-mannanase	3.2.1.78	1
GH135	Polysaccharides	α-1,4-galactosaminogalactan hydrolase	3.2.1.-	4
PL1	Pectin (homogalacturonan)	Pectate lyase	4.2.2.2	21
PL3	Pectin	Pectate lyase	4.2.2.2	10
PL4	Pectin (rhamnogalacturonan I)	Rhamnogalacturonan lyase	4.2.2.-	8
PL9	Pectin	Pectate lyase	4.2.2.2	2
		Exopolygalacturonate lyase	4.2.2.9	
PL11	Pectin	Rhamnogalacturonan endolyase	4.2.2.23	2
PL22	Pectin	Oligogalacturonate lyase	4.2.2.6	1
PL24	Pectin	Ulvan lyase	4.2.2.-	1

^a^ *CE* Carbohydrate esterases, *GH* Glycoside hydrolases, *PL* polysaccharide lyases

**Table 4:** Classes of auxiliary activity (AA), carbohydrate-binding module (CBM), and glycosyl-transferase (GT) enzymes in the genome of *Phomopsis longicolla* isolate MSPL 10–6

CAZy^a^ family	Description	Copy Number
AA1	Multicopper oxidases	6
AA2	Lignin peroxidase	16
AA3	Glucose-methanol-choline (GMC) oxidoreductases	75
AA4	vanillyl-alcohol oxidase	7
AA5	radical-copper oxidases	3
AA6	1,4-benzoquinone reductases	1
AA7	Glucooligosaccharide oxidase	96
AA8	Iron reductase	7
AA9	Copper-dependent lytic polysaccharide monooxygenases	35
AA11	monooxygenase	10
AA12	The pyrroloquinoline quinone-dependent oxidoreductase activity was demonstrated for the CC1G_09525 protein of *Coprinopsis cinerea*	2
AA13	Monooxygenase	1
CBM1	Cellulose-binding	21
CBM6	Amylase	1
CBM13	Cellulose-binding	2
CBM18	Chitin-binding	12
CBM20	Starch-binding	6
CBM21	Starch-binding	1
CBM23	Mannan-binding	1
CBM24	Alpha-1,3-glucan (mutan)-binding	12
CBM32	Binding to LacNAc (beta-D-galactosyl-1,4-beta-D-N-acetylglucosamine)	2
CBM35	Xylan-binding	4
CBM37	Xylanase	1
CBM42	Binding to arabinofuranose	1
CBM43	Beta-1,3-glucan binding	2
CBM48	Amylase	1
CBM50	Peptidoglycan-binding (LysM domain)	37
CBM63	Cellulose-binding	1
CBM66	β-fructosidase	2
CBM67	L-rhamnose-binding	6
GT1	UDP-glucuronosyl-transferase	12
GT2	Cellulose/chitin synthase	16
GT3	Glycogen synthase	1
GT4	Sucrose synthase	7
GT5	Glycogen glucosyltransferase	4
GT8	Lipopolysaccharide glucosyl-transferase	6
GT15	α-1,2-mannosyl-transferase	5
GT17	β-1,4-N-acetyl-glucosaminyl-transferase	1
GT20	α,α-trehalose-phosphate synthase	3
GT21	Ceramide β-glucosyl-transferase	3
GT22	Man6GlcNAc2-PP-Dol α-1,2-mannosyl-transferase	4
GT24	Glycoprotein α-glucosyl-transferase	1
GT25	Lipopolysaccharide beta-1,4-galactosyltransferase	6
GT28	Digalactosyl-diacyl-glycerol- synthase	1
GT31	Fucose-specific β-1,3-N-acetylglucosaminyl-transferase	4
GT32	α-1,6-mannosyl-transferase	13
GT33	Chitobiosyl-diphosphodolichol β-mannosyl-transferase	1
GT34	α-1,2-galactosyl-transferase	2
GT35	Starch phosphorylase	1
GT39	Protein α-mannosylt-ransferase	3
GT41	Beta-N-acetylglucosaminyltransferase	1
GT48	1,3-β-glucan synthase	1
GT50	α-1,4-mannosyl-transferase	1
GT55	GDP-Man: mannosyl-3-phosphoglycerate synthase	2
GT57	α-1,3-glucosyl-transferase	3
GT58	Man5GlcNAc2-PP-Dol α-1,3-mannosyl-transferase	1
GT59	Glc2Man9GlcNAc2-PP-Dol α-1,2-glucosyl-transferase	1
GT61	Xylanase	1
GT62	α-1,2-mannosyl-transferase	3
GT66	dolichyl-diphospho-oligosaccharide-protein Glycotransferase	1
GT68	O-alpha-fucosyltransferase	1
GT69	α-1,3-mannosyl-transferase	4
GT71	α-mannosyl-transferase	8
GT76	α-1,6-mannosyl-transferase	1
GT77	Xylanase	1
GT90	Xylanase	9
GT92	Glycanase	1

^a^ Carbohydrate-activated enzymes

### Repetitive elements and transposase

Classification of the repetitive elements can be generally divided into two classes: Class I elements (Retrotransposons) and Class II elements (DNA Transposons). Of 12,322 repetitive elements identified in the genome of the MSPL 10–6 isolate**,** 7036 (57.1%) of the repetitive elements were Class I, while 5249 (42.6%) belonged to Class II. There were 370 (0.3%) unknown/unclassified repetitive elements (Table 5). The major transposons were DNA/TcMar-Fot1 (41.8%), LTR/ Copia (30.7%), and LTR/Gypsy (24.4%) like.

**Table 5:** Classification of repetitive elements identified in the genome of *Phomopsis longicolla* isolate MS 10–6

	Total length (bp)	Repetitive content (%)	Genome content (%)
Class I (Retrotransposon)			
LTR retrotransposon^a^			
LTR/Copia	2,408,959	30.71	3.89
LTR/Gypsy	1,914,239	24.40	3.09
LTR/Other	80,954	1.03	0.13
Subtotal	4,404,152	56.14	7.11
Non-LTR retrotransposon			
LINE^b^	74,280	0.95	0.12
Total Class I	4,478,432	57.09	7.23
Class II (DNA transoposon)			
DNA/TcMar	3,277,211	41.77	5.29
DNA/Other	65,709	0.84	0.11
Total Class II	3,342,920	42.61	5.4
Other			
Satellites, rRNA, Unknown repeats	23,808	0.30	0.04
Total	7,845,160		12.67

^a^long terminal repeat retrotransposon

^b^long interspersed nuclear elements

### Horizontal gene transfers

**Fig. 4:**
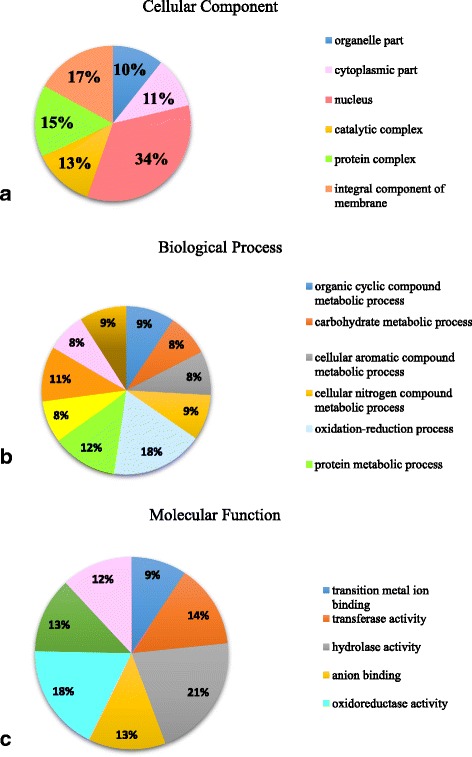
Distribution of annotated horizontal transferred genes in the genome of *Phomopsis longicolla* isolate MSPL 10–6 based on gene ontology analysis (**a**) cellular component, (**b**) biological process, and (**c**) molecular function

A total of 510 potential horizontal gene transfers (HGTs) were identified in the genome of the MSPL 10–6 isolate (Additional file 2: Table S2). They were originally from 53 species including 22 fungi, 26 eubacteria and 5 archaebacteria (Table 6). The majority of HGTs were from fungal origins (85.3%), while 13.3% and 1.4% of the HGTs were from eu- and archae- bacterial origins, respectively. Results of annotation of the HGTs based on gene ontology analysis are shown in Fig. 4. Over 70% of the HGTs were related to molecular functions.

**Table 6:** The donor, taxonomy, and number of proteins encoded by the horizontal transferred genes in in the genome of *Phomopsis longicolla* isolate MSPL 10–6

Donor	Taxonomy	Number of Proteins
*Acidobacteriales*	Bacteria	1
*Agaricales*	Fungus	1
*Auriculariales*	Fungus	4
*Bacillales*	Bacteria	5
*Boletales*	Fungus	2
*Botryosphaeriales*	Fungus	24
*Burkholderiales*	Bacteria	8
*Capnodiales*	Fungus	28
*Chaetothyriales*	Fungus	89
*Corynebacteriales*	Bacteria	5
*Cytophagales*	Bacteria	2
*Deinococcales*	Bacteria	1
*Dictyosteliida*	Fungus	1
*Dothideales*	Fungus	12
*Enterobacterales*	Bacteria	2
*Eurotiales*	Fungus	73
*Haemosporida*	Parasites	1
*Helotiales*	Fungus	66
*Hymenochaetales*	Fungus	1
*Lactobacillales*	Bacteria	1
*Micrococcales*	Bacteria	4
*Micromonosporales*	Bacteria	1
*Myxococcales*	Bacteria	3
*Nostocales*	Bacteria	1
*Oceanospirillales*	Bacteria	1
*Onygenales*	Fungus	8
*Orbiliales*	Fungus	5
*Oscillatoriales*	Bacteria	1
*Peniculida*	Oligohymenophorea	1
*Planctomycetales*	Bacteria	1
*Pleosporales*	Fungus	78
*Polyporales*	Fungus	3
*Pseudomonadales*	Bacteria	1
*Pseudonocardiales*	Bacteria	4
*Rhizobiales*	Bacteria	6
*Rhodobacterales*	Bacteria	1
*Rhodospirillales*	Bacteria	2
*Russulales*	Fungus	2
*Schizosaccharomycetales*	Fungus	1
*Sphingobacteriales*	Bacteria	2
*Sphingomonadales*	Bacteria	5
*Sporidiobolales*	Fungus	1
*Streptomycetales*	Bacteria	7
*Streptosporangiales*	Bacteria	1
*Sulfolobales*	Crenarchaeota	1
*Thermoproteales*	Crenarchaeota	1
*Tremellales*	Fungus	4
*Trichomonadida*	Parabasalia	3
*Venturiales*	Fungus	9
*Verrucariales*	Fungus	8
*Verrucomicrobiales*	Bacteria	1
*Xanthomonadales*	Bacteria	1
*Xylonomycetales*	Fungus	15
Total		510

## Discussion

In this study, the general genome feature of *P. longicolla* isolate MSPL10–6 from Mississippi, USA was described. The assembly of the genome was the result of combining the output from analyzing the sequences from both paired end and mate pair libraries. A de novo nuclear genome assembly was generated and characterized. The *P. longicolla* genome was estimated to be approximately 62 Mb, using both kmer and read coverage analyses. The size of the genome of *P. longicolla* MSPL 10–6 isolate appeared larger than other reported ascomycete soybean pathogens, such as *Diaporthe aspalathi* (55 Mb) [29], *Fusarium virguliforme* (50.5 Mb) [30] and *Macrophomina phaseolina* (49.3 Mb) [31]. The overall number of predicted gene in *P. longicolla* was 16,597, while it was 14, 962, 14,845, and 14,249 in *D. aspalathi* [29], *F. virguliforme* [30], and *M. phaseolina* [31], respectively. It is unknown whether the larger size of the genome and the bigger number of the predicted gene contributed in part to the fungal specialization and the pathogenicity of *P. longicolla* on soybean. Both *P. longicolla* and *D. aspalathi* are the members of *Diaporthe-Phomopsis* complex causing soybean diseases. They have similar culture morphology and very close relationships in taxonomy. However, *P. longicolla* is the primary cause of Phomopsis seed decay, while *D. aspalathi* primarily causes canker on soybean stem. It has been reported that soybean seeds, instead of stems and other soybean tissues, are more susceptible to *P. longicolla* than to other *Diaphorthe* species [14]. A comparative genome analysis is underway to address the questions about the pathogenicity mechanisms of those two species (Li et al., unpublished).

To investigate the genetic basis of pathogenicity, plant cell wall degrading enzymes (PCWDEs) in *P. longicolla* were identified and annotated. Results here indicated that *P. longicolla* contained abundance of genes encoding PCWDEs, which include all six classes of CAZys. For enzymes in the class of carbohydrate esterases (CE), the genome of *P. longicolla* contained 105 GH10 like genes that are absent in other pathogens, like *F. virguliforme* [32]. Plant cell walls contain abundant cellulose. Most of cellulose-degrading enzymes are classified into the glycoside hydrolase (GH) class. As indicated in Table 3, the substrates for the GH1, GH3, and GH5 degrading enzymes included not only cellulose, but also hemicellulose and pectin. It has been reported that most of biotrophic fungi do not have GH1 [33], while the genome of *P. longicolla* encodes six GH1 genes. Notably, there are 23 GH3 and 27 GH5 genes in the *P. longicolla* genome. Both GH3 and GH5 were common in pathogenic oomycetes, hemibiotrophic and necrotrophic fungi, which have more genes than biotrophic fungi and *Pythium* species [32]. The polysaccharide lyases (PL) are one of the important classes of PCWDEs. The PL class specializes in pectin degradation. It is well-known that pectin is the most divergent component of plant cell walls with different modifications on the side chains. The microbial pectinolytic enzymes have been studied and reviewed [34]. PL1 and PL3 were the most common pectin lyases found in plant pathogens. In this study, there were 21 and 10 copies of PL1 and PL3 in *P. longicolla*, respectively. Three other PL members, PL9, PL 20, and PL22, have been thought previously to be unique to *N. haematococca* and *F. virguliforme*, and were not found in other plant fungal pathogens or oomycetes [32], but both PL9 and PL22 domains were present in the *P. longicolla* genome. *F. graminearum* [35], *F. fujikuroi* [36], and *F. virguliforme* [32], *P. longicolla* had PL1, PL3, PL4, PL9, and PL22. They all had PL11 and PL24, except *F. virguliforme* [32]. However, *P. longicolla* did not have PL20 as *F. virguliforme* had.

Significantly, a novel fungal ulvan lyase (PL24; EC 4.2.2.-) was found in the *P. longicolla* genome. Ulvan lyases degrade ulvan, an anionic polysaccharide. This enzyme has only been reported recently, as the first members of a new polysaccharide lyase family in bacteria [37]. Ulvan is the most abundant component of the green algal cell wall. The role of ulvan lyases in the pathogenicity of *P. longicolla* is unknown. It will require further investigation. The PCWDE is one of the most important factors associated with pathogenicity of fungal pathogens. It could play a crucial role in infecting plants and influencing host resistance. The list of PCWDEs identified in *P. longicolla* laid the foundation for dissecting the mechanisms of the fungal pathogenicity through further functional analyses of genes encoding PCWDEs. Those analyses should aid in developing new strategies for breeding for resistance to Phomopsis seed decay in soybean.

To examine the genome architecture of *P. longicolla*, repetitive elements were analyzed. As noted, members of both class I and class II repetitive elements were found in the genomes of filamentous fungi [38]. Approximately 13% of the *P. longicolla* genome consists of repetitive elements. This is greater than the 10% and 1% in other ascomycetes *Magnaporthe oryzae* and *M. poae* genomes, respectively [39–41]. The majority (57.1%) of repetitive elements in the *P. longicolla* genome are categorized as Class I elements (retrotransposons). They are transcribed from DNA to RNA, and the RNA produced is then reverse transcribed into DNA. Moreover, retrotransposons mobilize via a “copy-and-paste”, which allow for many copies to be inserted throughout the genome. Thus, retrotransposons are the most common transposon in eukaryotes [42, 43] including *P. longicolla.* In addition, the long terminal repeat (LTR) is one of the main groups of retrotransposons [44]. The two main superfamilies of LTR retrotransposons found in fungi are Gypsy and Copia. In *P. longicolla* there were 30.7% of LTR/Copia and 24.4% of LTR/Gypsy among the repetitive elements. Since there are abundances of LTR/Copia present in the genomes of plants, fungi, animals, algae and several protists, it has been proposed that the ancestors of the LTR/Copia family probably co-existed with the ancestors of LTR/Gypsy before the separation between plants and other kingdoms [45].

The DNA transposons (class II elements) mobilize via a cut-and-paste mechanism that use a DNA intermediate, in which, the DNA itself is excised from the genome and integrated elsewhere. Mariner-like elements are one of prominent classes of the DNA transposons found in multiple species, including humans. This Class II transposable element is known for its uncanny ability to be transmitted horizontally between many species [46, 47]. It estimated that there were 14,000 copies of mariner in the human genome encompassing 2.6 million base pairs [48]. The first mariner-element transposons outside of animals were found in *Trichomonas vaginalis*, the most common pathogenic protozoan infection of humans [49]. Interestedly, the most major transposon in the *P. longicolla* genome was DNA/TcMar-Fot1 (41.8%). The function of this transposon in *P. longicolla* is unknown.

In the past, many documented HGT events inferred to fungi involved bacterial donors. For example, in a search for eubacterial-derived HGTs in 60 fully sequenced fungal species, Marcet-Houben and Gabaldon detected 713 transfer genes from bacteria [50]. Gene transfer between fungi has already been reported [51]. In a comparative genomic study of *Fusarium* species, four of *F. oxysporum’*s 15 chromosomes inferred to have been acquired through HGT from a fungal source [52]. Notably, chromosome 14, which is essential for pathogenicity of tomato, could be transferred between pathogenic and non-pathogenic strains of *F. oxysporum* resulting in conversion of non-pathogenic strains into pathogenic strains. Here, the majority of HGTs in the *P. longicolla* genome were of fungal origin (85.3%), while only 13.3% of the HGTs were from bacteria. Almost half of the HGT genes in the *P. longicolla* genome were related to molecular function. Further research will be necessary to address many open questions such as the impact of HGTs on the genome structure, gene function, and pathogenicity of *P. longicolla.*

The genome of the isolate MSPL10–6 was the first reported genome sequence in the fungal *Diaphothe-Phomopsis* complex causing soybean diseases. Our study represents the first genomic effort to discover the genome structure of *P. longicolla.* The genome data provide new insights into the gene repertoire and physiological potential of seed-borne pathogens. Additionally, the genomic resources presented here, including the genome sequences and annotations, detail lists of cell wall degrading enzymes, repetitive elements and horizontal transferred genes, enhance our knowledge about the biology and genetics of *P. longicolla.* These discoveries will facilitate further research on the genomic basis and pathogenicity mechanism of *P. longicolla,* and aid in development of improved strategies for efficient management of this pathogen.

## Conclusions

Phomopsis seed decay of soybean is one of the most devastating diseases affecting soybean seed quality worldwide. However, genomic basis and mechanism of the pathogenicity of *P. longicolla* on soybean was lacking. The draft genome of the *P. longicolla* isolate MSPL10–6 represents the first reported genome sequence in the fungal *Diaporthe-Phomopsis* complex causing soybean diseases. The MSPL 10–6 genome contains a number of unique genomic features, including an abundance of genes encoding cell-wall degrading enzymes, numerous repetitive elements, as well as horizontal transferred genes from eubacteria, fungi and archaebacteria. Information obtained from this study enhances our knowledge about the biology and genetics of the seed-borne pathogen will facilitate further research on the genomic basis and pathogenicity mechanism of *P. longicolla.* The study will aid in development of improved strategies for efficient management of this pathogen.

## Methods

### Isolation, identification, and cultivation of *P. longicolla* isolate

A *P. longicolla* isolate MSPL10–6 was isolated from field-grown soybean seed in Mississippi, USA in 2010 using the seed plating method. Briefly, over 100 randomly chosen soybean seeds that were harvested from the field were surface-disinfected in 0.5% sodium hypochlorite for 3 min, rinsed in sterile distilled water 3 times (3 min each time), and then placed on potato dextrose agar (Difico Laboratories, Detroit, MI) that was acidified (pH 4.8) with 25% lactic acid after autoclaving (APDA). Five seeds were plated on each 100 mm-diameter Petri dish. After 4 days of incubation at 24 °C in the dark, putative/potential *P. longicolla* was isolated, streaked to the new APDA plates and incubated under 12-h light-and dark cycles. After 4–6 days, monoconidial cultures were obtained.

Identification of *P. longicolla* was first based on morphological characteristics according to Hobbs et al. [1] and then was confirmed by analysis of the ITS region of rDNA amplified by PCR with primers ITS1, 5′-TCCGTAGGTGAACCTGCGG-3’and ITS4, 5′-TCCTCCGCTTATTGATATGC-3′ [53] and the translation elongation factor 1-α gene primer set EF1-728F, 5′- CAT CGA GAA GTT CGA GAA GG -3′, and EF1-986R, 5′-TAC TTG AAG GAA CCC TTA CC -3′ [54, 55]. Pathogenicity tests were performed using a cut-seedling inoculation method as described by Li et al. [56]. Isolate MSPL 10–6 was one of the most aggressive isolates causing severe soybean stem lesion in the greenhouse tests (data not shown). This isolate has also been used to screen soybean germplasm and successfully identified 23 new sources of resistance to PSD [17, 57].

### Genomic DNA extraction and sequencing

For DNA extraction, mycelial plugs (3-mm in diameter) from the margin of a 10-day old culture of MSPL 10–6 on APDA were cut and placed in potato dextrose broth (Difico Laboratories, Detroit, MI). After 4 days of incubation at 24 °C under 12-h light-and dark cycles, mycelia were collected on sterile cheesecloth, washed with sterile water, immediately frozen with liquid nitrogen, and lyophilized with a freeze-drier (IMC Instruments, Inc., Wisconsin, USA). Fungal mycelia were ground with a mortar and pestle and pulverized in liquid nitrogen. The genomic DNA was extracted using a Qiagen DNeasy Plant Mini Kit (Qiagen Inc., Valencia, CA) following the manufacturer’s instruction and qualified with Nanodrop (Thermo Scientific, Waltham, MA, USA).

Genomic DNA of the *P. longicolla* MSPL10–6 isolate was used to generate sequencing libraries as previous described [28]. Briefly, paired-end libraries were made with the TruSeq DNA PCR-Free Sample Preparation kit (Illumina San Diego, CA), while the no-gel mate-pair libraries were generated with the Nextera Mate-Pair Sample Preparation kit (Illumina San Diego, CA) according to the manufacturer’s protocols. All libraries were sequenced in separate lanes on an Illumina HiSeq 2500 sequencer using a TruSeq SBS sequencing kit (version 3, Illumina) at the Genomics Core Facility, Purdue University, West Lafayette, IN.

### De novo genome assembly

Adapter sequences and poor quality bases (Phred score < 20) for each sequence read were trimmed using the FASTX-Toolkit (http://hannonlab.cshl.edu/fastx_toolkit/index.html). A total of 72,216,734 mate-pair reads with a total of 8.2 billion bp representing 128-fold coverage, and 63,763,666 paired-end reads with a total of 6.2 billion bp, representing 97-fold coverage, were generated. The *P. longicolla* genome was assembled from both libraries using the software SOAPdenovo assembler version 2.04 [58]. Raw sequence data was deposited into NCBI’s SRA database, under accession number: AYRD00000000 (1) (http://www.ncbi.nlm.nih.gov/nuccore/AYRD00000000).

### Gene prediction and annotation

Gene prediction analysis was performed using a combination of homology searching and de novo prediction using Augustus web server [59, 60] with complete gene option enabled and default for the rest of the parameters*. F. graminearum* [31] was used as the reference species due to the relatively close phylogenetic relationship to *P. longicolla* MSPL10–6 among the genome sequences available in GenBank.

Predicted genes were functionally annotated using Blast2GO [61]. The gene models were BLAST-ed (BLASTx) [62] against the NCBI non-redundant protein database. Then domain finding searches were done using InterProScan [63]. Enzyme codes and GO ontologies were then assigned to the gene models as described by the Gene Ontology Consortium (http://geneontology.org).

### Identification of carbohydrate-activated enzymes (CAZys) in the *P. longicolla* genome

To identify CAZys in the genome of *P. longicolla* isolate MSPL 10–6, Augustus program (http://bioinf.uni-greifswald.de/webaugustus/prediction/create) [59] trained with the parameters of the species *Fusarium graminearum* was used to predict putative proteins of the CAZy family. Using the web resources of dbCAN, CAZy domains in the genome of *P. longicolla* were identified with a cutoff E value of 10^−3^ [64]. Classification of CAZy was conducted as described by Chang et al., 2016 [32].

### Identification and classification of repeat elements in the *P. longicolla* genome

Repeat elements were identified using two well-cited software packages, RepeatMasker (v. 4.0.5) and Censor (v. 4.2.29) [65, 66]. The analysis was partially carried out at the Bioinformatics Core at Purdue University. For RepeatMasker, the optimized default parameters were utilized with the ‘-lib’ option to find repeats associated with the RepBase file (v. 20.03) above. Additionally, the ‘-species Fungi’ option was also used in a separate analysis to find fungal repeats based on RepBase-derived libraries provided by RepeatMasker. Similarly, for Censor, with the ‘censor.ncbi’ module, the optimized default parameters were utilized and the ‘-lib’ option to find repeats.

The predicted protein sequences were compared against the NCBI non redundant database using BLASTP to find the top hits. The genes from contigs larger than 500 bp were used since the same contigs were used for repeat prediction. Similarly, the predicted protein sequences were compared against protein sequences of the closest relative (*Diaporthe ampelina*) by creating a BLAST-able database***.*** The combined annotation file containing the annotation of each predicted genes and *D. ampelina* database was generated using in house scripts.

### Identification of horizontal gene transfers

To identify putatively horizontally transferred genes in the *P. longicolla* isolate MSPL 10–6, the HGTector software (http://bmcgenomics.biomedcentral.com/articles/10.1186/1471-2164-15-717) was used, which follows a hybrid approach between “BLAST-based” and phylogenetic. The software was setup with the following stringency parameters: threads = 12, selfTax = 1,230,121, closeTax = 147,550, searchTool = blastp, e-value cutoff = 1 × 10^−5^ for the BLAST hits, and default values for the rest of the parameters. The *P. longicolla* isolate MSPL 10–6 predicted proteins were blasted against a local NCBI nr database. NCBI Taxonomy database (downloaded on February 20, 2017) was used to classify BLAST matches.

## Additional files

Additional file 1: Table S1. A list of putative genes encoding cell wall degrading enzymes. (TXT 73 kb)

Additional file 2: Table S2. A list of horizontal transferred genes identified in the genome of *Phomopsis longicolla* isolate MSPL 10–6. (XLSX 75 kb)

## Abbreviations

AA: Auxiliary activity
CAZy: Carbohydrate-activated enzymes
CBM: Carbohydrate-binding module
CE: Carbohydrate esterases
GH: Glycoside hydrolases
GO: Gene ontology
GT: Glycosyl-transferase
HGT: Horizontal gene transfer
LINE: Long interspersed nuclear elements
LTR: Long terminal repeat
PCWDE: Plant cell wall degrading enzyme
PL: Polysaccharide lyases
PSD: Phomopsis seed decay
